# Public-Public Collaboration for Food Safety Risk Management: Essence, Modes, and Identification of Key Influencing Factors Using DANP

**DOI:** 10.3389/fpubh.2022.944090

**Published:** 2022-07-13

**Authors:** Liwei Zhang, Ke Qin, Yufeng Li, Linhai Wu

**Affiliations:** ^1^College of Economics and Management, Shanghai Ocean University, Shanghai, China; ^2^School of Business, Jiangnan University, Wuxi, China

**Keywords:** food safety risk, cross-boundary governance, public-public collaboration, DANP, key factor

## Abstract

**Objective:**

Food safety risk management is an important cross-boundary issue from both theoretical and practical standpoints. Because food safety has the social attributes of public goods, public-public collaboration can be considered a particularly important mode of cross-boundary governance. The study aims to provide a theoretical basis for the Chinese government to promote public-public collaboration for food safety risk management by identifying key factors.

**Methods:**

Based on a review of literature across diverse fields, such as political science, sociology, and new public governance, this study discusses the essence, modes, and dilemma of public-public collaboration for food safety risk management using practical explorations in various countries as the main thread and taking into account the actual situation in China. Moreover, this study quantitatively analyzes the relationships between the dimensions and factors affecting public-public collaboration and identifies key dimensions and factors using the Decision-making Trial and Evaluation Laboratory-based Analytic Network Process (DANP).

**Results:**

Among the 20 factors in the calculation results of DANP, Lawmaking has the highest value of (*f*_*i*_+*e*_*i*_) (7.022) and ranks sixth in terms of influence weight. The (*f*_*i*_+*e*_*i*_)value of Professionalism (6.993) ranks second and its influence weight ranks fourth. The (*f*_*i*_+*e*_*i*_) value of Administrative enforcement (6.722) ranks fifth, and its influence weight ranks seventh. The (*f*_*i*_+*e*_*i*_) value of Improvement of the social environment (6.699) ranks sixth, and its influence weight ranks fifth. The (*f*_*i*_+*e*_*i*_) value of Legal authorization (6.614) ranks seventh, and its influence weight ranks tenth. Data analysis indicated that these are the five key factors affecting the governance capacity in public-public collaboration for food safety risk management.

**Conclusion:**

The legal basis is the most important dimension affecting public-public collaboration. Legislation-based governance, administrative law enforcement–based governance, and social environment improvement–based governance in the behavior and capabilities dimension, professionalism in the basic characteristics dimension, and laws and regulations in the legal basis dimension are the five key factors.

## Introduction

Since the global outbreak of COVID-19, food safety risk management has become more complex (1), and cross-boundary governance, including public-public, public-private, multiagent, interregional, and even cross-border collaboration, has become increasingly prominent. Because food safety has the social attributes of public goods, public-public collaboration is applicable as a particularly important mode of cross-boundary governance. However, most countries have not effectively addressed the problem of fragmentation among government regulators (2). Numerous studies have examined the main factors affecting public-public collaboration and yielded important findings. However, these findings are dispersed across different fields in the literature. There are few systematic studies on this topic.

Accordingly, this study summarizes relevant literature from diverse fields such as political science, sociology, and new public governance. On this basis, using practical explorations of public-public collaboration for food safety risk management in various countries as the main thread, and taking into account the actual situation in China, quantitative research is conducted using DANP to systematically examine the relationship between the dimensions and factors affecting public-public collaboration on food safety risk management and identify key dimensions and factors. This study presents systematic and comprehensive research results and provides guidance for policy-making for promoting public-public collaboration in China.

## Essence and Modes of Public-Public Collaboration for Food Safety Risk Management

Food safety risks exist in all stages of human social development, and managing food safety risks has always been a major public issue facing all countries throughout the world (3). However, the disadvantages of Western governments playing the role of “super nanny” in public management have become increasingly apparent since the 1960s. The contradictions in the management of public affairs, such as employment, social security, public security, environmental protection, food safety, and maintaining fair market competition, have become increasingly prominent, making it difficult for governments to effectively manage major public issues (4). As a result, the New Public Management reform occurred, which started in the UK, then took place in the US, subsequently extended to other western developed countries, and finally spread to many developing countries (5). However, problems such as inequality of politics and resources, fragmentation of power, and cleavability of services became more prominent in Western countries after nearly 20 years of New Public Management reform (6). Hence, Western countries experienced a second reform known as the post- New Public Management in the late 1990s. The two reforms are considered landmark events of public management reform by Western governments. It has been recognized that public management is an important cross-boundary theoretical and practical issue. The concept and theory of cross-boundary governance have thus been gradually developed and applied in practice, which led governments to effectively promote the development of public-public collaboration for food safety risk management (7).

### Essence of Public-Public Collaboration

Food safety is characterized by inseparability of utility, non-rivalry of consumption, and non-exclusivity of benefits (8). Moreover, food safety risks travel along the supply chain. Due to the butterfly effect, the possible superposition and resonance of risks during such travel may trigger food safety incidents (9). Food safety incidents not only harm public health, but also affect the development of the food industry and even cause social unrest (10). Therefore, all countries face the challenge of preventing food safety risks (11). Many countries' governments have devoted significant efforts and implemented a series of measures to address this issue (12). However, as the New Public Management and post-New Public Management reforms proceeded, it was realized that, similar to other public affairs, food safety risk management is a task that requires multiagent collaboration (13) to break through the inherent barriers between public sectors in traditional public management, resolve the problems of blurred boundaries, overlap, and fragmentation, and eliminate government and market failures caused by the blurred functional boundaries and fragmentation among the various actors (14). This is the essence of public-public collaboration for food safety risk management.

### Main Modes of Public-Public Collaboration

Since the 1990s, the concept of cross-boundary governance in the public governance theory of Western countries has been extended to apply to food safety risk management. A variety of public governance theories, such as networked governance, multilevel governance, collaborative governance, and whole of government (15–19), have become important theoretical paradigms to guide research on cross-boundary governance for food safety risk management. Based on the actors involved, Toppinen and Korhonen (20), Bunthof et al. (21), Yu and Xiao (22), Kim et al. (23), and Diehlmann et al. (24) defined four basic types of cross-boundary governance of public affairs, including food safety risk management: public-public, public-private, interregional, and multiagent. Regardless of type, however, the essence of cross-boundary governance is that different actors at the same or different levels achieve coordinated governance of major public affairs for the sake of the public interest by breaking through traditional boundaries (25).

As a commodity, food has common attributes of commodities. However, it also has the social attributes of public goods because food safety is related to the health of citizens (26). Therefore, public-public collaboration is the most important mode of cross-boundary governance in this case. Similar to the governance of other public affairs, there are two modes of public-public collaboration for food safety risk management: horizontal collaboration, which crosses the boundaries of government agencies at the same level (27), and vertical collaboration, which crosses the boundaries of government agencies at different levels (25). All other modes are a combination or extension of these two modes.

From a global perspective, although different countries have different structures of government agencies, food safety risk management always involves not only specific industry regulators, such as those governing the agriculture, forestry, fishery, and processing industries, but also public service agencies, such as those overseeing market regulation, health, quality standards, food consumption, and imports and exports. Therefore, risk prevention requires government agencies at the same level to cross organizational boundaries to overcome fragmented governance (28), as well as building a clear governance authority and responsibility system across government agencies from the central to the local level to give full play to their respective advantages, share information, effectively interact, and ultimately achieve coordinated and unified efficient governance (29).

### Dilemma of Public-Public Collaboration

For a long time, the governments of many countries, especially those of Western countries, have conducted extensive practical explorations regarding how to enact food safety regulators and achieve effective cross-boundary governance among regulators. The US federal government's food safety regulator was established in 1906. At present, there are as many as 15 regulators at the US federal level that perform different functions. Although public-public collaboration has been continuously optimized in long-term development, the problems of fragmentation, inconsistency, and overlap of regulation among government agencies have not been well-resolved, which has affected the overall governance capacity (30).

In fact, these problems are common in Western countries, although to varying degrees (31). In response, there have been calls in Western countries over the last 20 years to merge regulators to eliminate gaps between them. In the US, it has been suggested that the federal government should merge multiple regulators into a single one, which is believed to be the most effective way to address the fragmentation of regulation among various agencies (32). However, the merger of regulators involves a series of complex issues, such as legal revision, resource reorganization, and system reconstruction. Moreover, the food supply chain's complexity, which is a result of factors such as a long industrial chain, low ignition point, and many contacts, the cross-boundary governance among regulators may be more effective than a simple merger (33). Regrettably, however, Western countries have yet to resolve the fragmentation among regulators, and cross-boundary governance still faces many intractable problems (2).

The same is true in China. Over the last 40 years of reform and opening up, the Chinese government has carried out eight different reforms of food safety regulators, with one reform occurring approximately every 5 years. Up to now, the regulatory structure has been based on the State Administration for Market Regulation, Ministry of Agriculture and Rural Affairs, General Administration of Customs, and National Health Commission, supplemented by more than 10 other participating agencies, such as Ministry of Commerce, State Administration of Grain, National Forestry and Grassland Administration, and Ministry of Education. However, a clear fragmentation of regulation still exists among these agencies, which is the inherent institutional reason for the continuous emergence of food safety problems in China, and thus requires further reforms.

## Dimensions and Factors Affecting Public-Public Collaboration for Food Safety Risk Management

A comprehensive review of existing literature on cross-boundary governance suggested that the major factors affecting the governance capacity in public-public collaboration for food safety risk management can be examined in terms of five dimensions: basic characteristics, legal basis, functions, behavior and capabilities, and infrastructure and culture of government agencies.

### Basic Characteristics of Government Agencies and Public-Public Collaboration

A country's government, consisting of different levels of legislative, executive, and judicial institutions, is the manifestation of the state's authority (34). The literature review revealed that government agencies exhibit four basic characteristic factors: legal person, power level, subordination, and professionalism (35). These are important dimensions affecting public-public collaboration. Ardoin et al. (36) suggested that legal person characteristics give government agencies the ability to perform civil responsibilities for public governance. The power-level characteristics refer to the administrative levels of agencies in the national governance system. The higher the power level, the higher the authority of governance, the stronger the ability to allocate resources and coordinate other agencies, and the greater the impact on public-public collaboration (37). For example, the US Federal Food and Drug Administration (FDA) is a specialized government agency that relies on the central government's authority to coordinate local governments to jointly ensure food safety (38).

The subordination characteristics refers to whether the agencies are in a vertical relationship of leading or being led by other agencies in the governance system, which affects the public-public collaboration. In the Chinese government's food safety risk management system, customs agencies responsible for import and export food safety regulations are in a typical vertical relationship. Local customs agencies are directly led by higher-level customs agencies and only have a synergistic relationship with local market regulators at the same level. In contrast, there is no subordination between the US federal and local FDAs, and local FDAs are independent agencies (39).

Professionalism refers to an agency's professional competence to manage food safety risks. Liu (31) investigated food safety regulators in 22 countries and found that despite their different configurations, they all met relevant professional requirements, such as having antitrust and quality control standards, and requiring quarantine of plants and animals. Woldesenbet (35) found that professional competence is an important factor affecting public-public collaboration. For example, the foundation of the US's global reputation for food safety lies in its long-term focus on professional capacity building of the FDAs at all levels.

### Legal Basis of Government Agencies and Public-Public Collaboration

Legal liability is a distinctive development that differentiates modern societies from traditional ones. Legal authorization acts as the basic guarantee for public-public collaboration for food safety risk management (40, 41). The 15 agencies set up by the US federal government related to food safety regulation are authorized by at least 30 laws (42). Different legal bases have specific applicability and binding force. Laws passed by the legislature are mandatory, whereas normative documents issued by the government are less binding (43). In China, central and local regulators usually implement non-legislative documents with a certain binding force within the scope of legal authorization, i.e., normative documents, to regulate important food safety matters.

Meanwhile, informal rules, including voluntary initiatives, although not mandatory, have become an important basis for mutual compliance among government agencies and promoting public-public collaboration due to the ambiguity of the law (44). Robinson (45) reported that 71 inter-agency agreements on food safety regulation have been signed among United States Department of Agriculture (USDA), FDA, Environmental Protection Agency (EPA), and the National Marine Fisheries Service (NMFS). Regarding some food safety regulation issues, such as food safety risk assessment and foodborne disease prevention, the US FDA, Food Safety and Inspection Service (FSIS), Centers for Disease Control and Prevention (CDC), and National Institutes of Health (NIH) have formed PulseNet, an inter-agency network to monitor, detect, and investigate outbreaks of foodborne disease outbreaks (46).

Path dependence refers to the fact that once a rule is formed in a system, it is often difficult to change the actual and potential impact of that rule. Fortwengel and Keller (47) found that path dependence exists for rules in public-public collaboration. Chen et al. (48) believed that the segmentation of regulations among agencies as long implemented in China is a typical manifestation of path dependence. Merrill and Francer (49) also suggested that path dependence exists in the US government's food safety regulation system, and that the historically formed division of powers among government agencies is particularly stubborn.

### Functions of Government Agencies and Public-Public Collaboration

Generally speaking, from the vertical perspective, central regulators deal with the most important administrative affairs in food safety risk management on behalf of the state and occupy a dominant position, whereas local regulators are in a subordinate position. From the horizontal perspective, the positions, functions, and responsibilities of various agencies at the same level in the food safety co-governance system do not match. For example, since the reform and opening up in 1978, China has undergone eight nationwide institutional reforms to develop a regulatory system based on the market regulation, agriculture and rural affairs, customs, and health agencies, supplemented by other participating agencies. Among them, the agriculture and rural affairs, health, and customs agencies are responsible for regulating the production of agricultural products prior to entering the market, development of food safety standards, and import and export food safety regulation, respectively. They are all single-function dominant agencies in this system.

The market regulation agency undertakes almost all functions other than those undertaken by the agriculture and rural affairs, health, and customs agencies, such as those concerning food production and processing, storage and circulation, and consumption, and thus is a comprehensive dominant agency. Other participating agencies, such as forestry, grain, commerce, and education agencies, are indirectly involved in governance in a specialized manner (50), and are thus all auxiliary agencies. According to resource scarcity theory, government agencies with different functions hold and accumulate different resources and play different roles in the governance system (51).

Compared with the situation in China, Western countries not only have different modes of multiagency food safety governance, but the functions, responsibilities, and authority of the relevant agencies are also different. For example, the USDA's authority is greater than that of the FDA (52). The same is true in the European Union (EU). Although there are multiple agencies for food safety management in EU countries, the European Food Safety Authority plays a dominant role (53).

### Behavior and Capabilities of Government Agencies and Public-Public Collaboration

Lawmaking, administrative enforcement, judicial enforcement, and improvement of the social environment are the main paths available for legislative, executive, and judicial agencies to achieve public-public collaboration for food safety risk management. However, the behavior of government agencies is determined by their legal authorization, but their governance capabilities in public-public collaboration inherently depend on their own capabilities (54). Boatemaa et al. (55) found that fragmentation of a legislature's legislative procedures from the administration's enforcement procedures restricted the effect of public-public collaboration. The research of Simon (56) on China's legal framework indicated that legislation should fully consider organically integrating the governance functions of legislative, administrative, and judicial agencies, which could help promote public-public collaboration.

Gazley (57) suggested that the legislature's legislative capacity affects public-public collaboration. Winders (58) argued that the effect of public-public collaboration depends on the organic combination and integration of the capabilities of participating agencies. In addition, based on the practice of many countries, the governance capacity for food safety risk management also depends on the improvement of the entire social environment, requiring not only the dominant agencies to perform their functions, but also the joint efforts of auxiliary agencies (59).

### Infrastructure and Culture of Government Agencies and Public-Public Collaboration

The governance capacity in public-public collaboration for food safety risk management also depends on the collaborative relationships among the government agencies, especially their ability to share governance resources and infrastructure conditions (60). If the chains of power, responsibility, and information between government agencies are integrated, information can be obtained, transmitted, and shared effectively, which contributes to public-public collaboration (61). Government agencies not only require information sharing, but also need to widely apply new technologies, such as information technology, which has become an important means of public-public collaboration in modern society (62).

Cultural factors also affect the implementation efficiency of formal or informal rules among government agencies (63). Reduced bureaucracy and an institutional culture with incentives or accountability will stimulate the internal motivation of agencies to participate in public-public collaboration (64). The higher the social concern about public affairs, the easier it is to generate external environmental pressure on public-public collaboration. With the continuous development of biotechnology, the deterioration of the ecological environment, and the continuous improvement of living standards and public scientific literacy, food safety will always be a public matter of high social concern, providing such a continuous external driving force for public-public collaboration (65).

## Identification Of Key Dimensions and Factors Affecting Public-Public Collaboration

Based on the above literature and the reality of China, a set of dimensions and factors that affect public-public collaboration for food safety risk management is defined as shown in Table 1.

**Table 1:** A set of dimensions and factors that affect public-public collaboration for food safety risk management is defined.

**Dimensions**	**Factors**
Basic characteristics of government	Legal person (d_1_)
agencies (D_1_)	Power level (d_2_)
	Subordination (d_3_)
	Professionalism (d_4_)
Legal basis of government agencies	Legal authorization (d_5_)
(D_2_)	Normative documents (d_6_)
	Informal rules (d_7_)
	Path dependence (d_8_)
Functions of government agencies	Comprehensive dominant agency (d_9_)
(D_3_)	Single-Function dominant agency (d_10_)
	Auxiliary agency (d_11_)
Behavior and capabilities of	Administrative enforcement (d_12_)
government agencies (D_4_)	Judicial enforcement (d_13_)
	Lawmaking (d_14_)
	Improvement of the social environment (d_15_)
Infrastructure and culture of	Information sharing (d_16_)
government agencies (D_5_)	Information technology (d_17_)
	Sectoral interdependence (d_18_)
	Institutional culture (d_19_)
	Social concern (d_20_)

### Methodology

Table 1 lists the five dimensions and 20 factors identified as affecting public-public collaboration for food safety risk management in the global context. These dimensions and factors are not independent, but interweave, influence, and interact with each other and thus constitute a complex system. Identifying the key dimensions and factors and determining how and to what degree they influence each other will undoubtedly be of great significance to explore ways to improve the governance capacity in public-public collaboration.

Decision-making Trial and Evaluation Laboratory (DEMATEL) is considered an effective method for determining the mutual influence of causal factor chains in complex systems (66), and it is usually used in conjunction with expert systems. The Analytic Network Process (ANP) was proposed by Saaty and Saaty (67) on the basis of Analytic Hierarchy Process (AHP), and is a method to determine the weight of indicators using the influence relationships between them (68). DANP is the combination of DEMATEL and ANP. It integrates the advantages of these two methods and can obtain the optimal solution of the complex relationship between multiple dimensions and factors in a complex system through computational science (69, 70). Therefore, DANP is used in the present study.

### Identifying the Influence Relationships Between Dimensions and Factors

The process used to identify the key dimensions and factors that affect public-public collaboration for food safety risk management using DEMATEL is as follows:

Step 1: Identify the dimensions and factors that affect the system. Based on the literature presented in Table 1, D_1_, D_2_, D_3_, D_4_, and D_5_ are used to represent the five dimensions of government agencies participating in public-public collaboration for food safety risk management, i.e., basic characteristics, legal basis, functions, behavior and capabilities, and infrastructure and culture. Then, d_1_, d_2_, d_3_, and d_4_ represent the four characteristic factors of D_1_, and d_5_, d_6_, d_7_, d_8_,..., d_16_, d_17_, d_18_, d_19_, and d_20_ are used to represent the characteristic factors of the dimensions D_2_, D_3_, D_4_, and D_5_, respectively.

Step 2: Investigate the relationship between factors. Using the expert opinion method, 19 experts, including 5 government officials in the market regulation system, 7 researchers from universities and research institutions, 2 experts of industry associations and 5 senior executives of food production enterprises, were invited to form an expert group. The pairwise relationships between the factors were scored according to the corresponding integer values in Table 2 to determine the importance of each factor in the system. The initial direct relation matrix *A* =_[*d*_*ij*_]20 × 20_ between factors was determined by regression estimation.


(1) $$\begin{array}{r} A=\left(\begin{array}{ccc} d_{11} & \cdots & d_{1\;20}\\ \vdots & \ddots & \vdots \\ d_{20\;1} & \cdots & d_{20\;20} \end{array}\right) \end{array}$$

where *d*_*ij*_ is the direct influence relationship between two factors, representing the direct influence of factor *i* on factor *j* (*i* and *j* = 1, 2, 3,...,20); for example, *d*_12_ indicates the direct influence of d_1_ on d_2_. To examine the mutual influence of two factors, if *i* = *j*, let *x*_*ij*_ = 0, indicating that the influence of each factor on itself is 0; that is, there is no influence. Hence, all elements on the main diagonal of the initial direct relation matrix A are recorded as 0.

**Table 2:** Conversion relationship between linguistic variables and integer values.

**Linguistic variables**	**Integer values**
No influence	0
Very low influence	1
Low influence	2
High influence	3
Very high influence	4

*Refer to Hsu et al. (71) and Lu et al. (72) for the design of linguistic variables and their corresponding integer values in the table*.

Step 3: Calculate the normalized direct influence coefficient and normalized influence matrix. The normalized direct relation matrix *G* (*G* = _[*g*_*ij*_]20 × 20_) is expressed by equation (2). The normalized direct influence coefficient *g*_*ij*_ in equation (3) represents the influence of factor *i* on factor *j*.


(2) $$\begin{array}{r} G=g_{ij}\times A \end{array}$$

(3) $$g_{ij}=\min\left\{\frac{1}{\underset{1\;\leq \;i\;\leq \;20}{\max}\sum_{j=1}^{20}d_{ij}},\frac{1}{\underset{1\;\leq \;j\;\leq \;20}{\max}\sum_{i=1}^{20}d_{ij}}\right\}$$

Step 4: Calculate the total relation matrix *T* between factors. *T* =_[*t*_*ij*_]20 × 20_ can be calculated by *T* = *G*+*G*^2^+*G*^3^+…*G*^*h*^ = *G*(*I*+*G*+*G*^2^+…*G*^*h*−1^)[(*I*−*G*)(*I*−*G*)^−1^] = *G*(*I*−*G*^*h*^)(*I*−*G*)^−1^where *I* is the identity matrix, *T*_*ij*_ represents the direct and indirect influences of factor *i* on factor *j*, and (*I*−*G*)(*I*−*G*)^−1^ = *I*. Therefore, when *h* → ∞, $D^{h}={\left[0\right]}_{n\times n}$, the total relation matrix *T* can be expressed by equation (4):

(4) $$T=G{\left(I-G\right)}^{-1}$$

Step 5: Calculate the influences given and received by each factor. Compute the row and column sums of matrix *T* to obtain the influences given and received by the corresponding factor, respectively. The following equations are used:


(5) $$\begin{array}{r} f_{i}=\overset{20}{\underset{j=1}{\sum }}t_{ij} \end{array}$$

(6) $$\begin{array}{r} e_{i}=\overset{20}{\underset{j=1}{\sum }}t_{ji} \end{array}$$

where *f*_*i*_ is the influence given by factor *i*, and *e*_*i*_ is the influence received by factor *i*.

Step 6: Calculate the centrality and causality of each factor. Centrality is obtained by adding the influences given and received by a system factor, which indicates a factor's position in the system as well as its effect size. The greater the centrality, the greater the factor's effect on public-public collaboration for food safety risk management. Causality is obtained by subtracting the influence given by the factor from that received by it. A positive causality indicates that the factor is a cause factor in the system, whereas a negative one indicates that the factor is an effect factor. The following equations are used:


(7) $$\begin{array}{r} m_{i}=f_{i}+e_{i} \end{array}$$

(8) $$\begin{array}{r} n_{i}=f_{i}-e_{i} \end{array}$$

where *m*_*i*_ is centrality, and *n*_*i*_ is causality.

Based on the scores given by the expert group for the importance of each factor, the initial direct relation matrix A shown in Table 3 is obtained according to the calculation method in Step 2.

**Table 3:** Initial direct relation matrix A between different influencing factors.

**Factors**	**d_**1**_**	**d_**2**_**	**d_**3**_**	**d_**4**_**	**d_**5**_**	**d_**6**_**	**d_**7**_**	**d_**8**_**	**d_**9**_**	**d_**10**_**
d_1_	0.000	1.000	0.636	2.273	2.000	2.000	2.000	1.455	0.727	0.636
d_2_	1.636	0.000	1.455	2.182	2.091	2.091	1.455	1.545	1.818	1.273
d_3_	1.636	0.818	0.000	1.545	1.909	1.818	1.091	1.455	1.818	1.364
d_4_	1.909	0.727	0.636	0.000	1.636	1.818	1.909	2.182	1.091	0.909
d_5_	2.091	1.455	1.182	2.182	0.000	2.182	1.727	2.455	1.909	1.909
d_6_	1.636	1.182	1.000	2.091	1.182	0.000	1.091	1.909	1.091	1.000
d_7_	1.000	0.636	0.273	1.909	0.727	0.727	0.000	1.364	0.636	0.636
d_8_	0.909	0.273	0.545	1.909	1.364	1.273	1.273	0.000	1.000	0.909
d_9_	1.455	1.000	1.545	2.182	1.182	1.273	1.364	1.636	0.000	0.727
d_10_	1.364	0.727	0.909	1.909	1.182	1.091	1.182	1.727	0.818	0.000
d_11_	0.727	0.727	1.000	1.364	0.818	0.636	1.182	1.182	0.455	0.273
d_12_	1.818	0.909	0.636	2.000	2.091	1.909	1.455	1.818	1.273	1.182
d_13_	1.545	0.727	0.545	1.909	2.182	2.091	1.364	1.364	1.000	0.909
d_14_	1.364	1.000	0.545	2.091	2.364	2.364	1.273	1.636	1.364	1.182
d_15_	1.091	0.818	0.545	1.727	1.455	1.818	1.909	1.091	1.091	0.909
d_16_	1.455	0.909	1.000	2.364	0.818	1.091	1.364	1.273	1.455	1.091
d_17_	1.273	0.545	0.545	1.818	0.909	1.000	1.364	1.455	1.273	1.000
d_18_	1.636	0.636	1.000	1.909	0.909	1.000	1.364	1.727	1.455	0.818
d_19_	1.455	0.636	0.818	2.364	0.909	1.000	1.636	1.273	1.091	0.818
d_20_	1.455	0.727	0.545	2.182	1.909	2.000	1.818	1.364	1.182	1.000
**Factors**	**d**_**11**_	**d**_**12**_	**d**_**13**_	**d**_**14**_	**d**_**15**_	**d**_**16**_	**d**_**17**_	**d**_**18**_	**d**_**19**_	**d**_**20**_
d_1_	0.364	2.455	1.818	1.909	1.636	2.545	1.818	2.000	2.545	1.727
d_2_	1.182	1.818	1.545	1.636	1.636	2.545	2.000	2.000	1.818	1.727
d_3_	1.455	2.182	1.818	1.909	1.455	2.000	1.818	1.818	1.545	1.364
d_4_	0.818	2.000	2.000	2.091	2.182	2.727	2.273	1.909	2.727	2.364
d_5_	1.545	2.818	3.182	3.273	2.818	2.273	2.273	1.818	1.636	1.727
d_6_	0.818	2.182	2.182	2.636	2.273	1.818	1.727	1.455	1.909	1.545
d_7_	0.545	0.818	0.909	1.273	1.727	1.364	1.182	1.091	1.727	1.818
d_8_	0.727	1.818	1.636	2.000	1.909	1.727	1.909	2.000	1.909	1.545
d_9_	0.636	2.000	1.818	1.909	2.182	3.000	2.636	2.727	2.000	2.182
d_10_	0.545	1.818	1.545	1.545	1.455	1.909	1.818	1.727	1.636	1.636
d_11_	0.000	1.273	1.000	1.091	1.000	1.364	1.455	1.545	1.364	1.727
d_12_	0.909	0.000	1.818	2.636	2.182	1.909	2.273	1.727	1.636	1.818
d_13_	0.727	1.727	0.000	2.636	1.909	1.727	2.000	1.545	1.545	1.636
d_14_	0.909	2.182	2.273	0.000	2.000	1.727	2.091	1.727	1.727	1.727
d_15_	0.727	1.636	1.727	2.182	0.000	1.636	2.091	1.818	2.000	2.636
d_16_	0.818	2.182	2.364	2.182	2.364	0.000	2.818	3.091	2.364	2.000
d_17_	0.727	2.273	2.273	2.364	2.273	3.182	0.000	2.545	2.364	2.091
d_18_	0.909	1.455	1.636	1.455	1.545	2.455	2.364	0.000	2.182	1.182
d_19_	0.636	1.636	1.909	1.909	2.545	1.818	1.727	2.000	0.000	1.909
d_20_	0.818	2.364	2.091	2.273	3.000	1.636	2.273	1.636	1.909	0.000

The initial direct relation matrix was normalized using equation (3) according to Steps 3 and 4, and then limits were computed using MATLAB according to equation (4) to obtain the total relation matrix *T* of different influencing factors. The Influence given and received by each factor affecting public-public collaboration for food safety risk management, denoted by *f*_*i*_ and *e*_*i*_, respectively, were calculated using equations (5, 6) according to Step 5. On this basis, the data set of (*f*_*i*_−*e*_*i*_, *f*_*i*_+*e*_*i*_) was calculated using equations (7, 8) according to Step 6, and the results of DEMATEL in Table 4 were obtained.

**Table 4:** Results of DEMATEL.

**Dimensions**	***f*_*i*_**	***e*_*i*_**	***f*_*i*_−*e*_*i*_**	***f*_*i*_+*e*_*i*_**	***Factors***	***f*_*i*_**	***e*_*i*_**	***f*_*i*_−*e*_*i*_**	***f*_*i*_+*e*_*i*_**
D_1_	1.265	1.111	0.154	2.376	d_1_	3.134	2.730	0.404	5.864
					d_2_	3.316	1.546	1.770	4.862
					d_3_	3.049	1.424	1.625	4.473
					d_4_	3.299	3.694	−0.395	6.993
D_2_	1.205	1.227	−0.022	2.432	d_5_	3.892	2.722	1.170	6.614
					d_6_	3.039	2.906	0.133	5.945
					d_7_	2.038	2.811	−0.773	4.849
					d_8_	2.642	2.921	−0.279	5.563
D_3_	1.185	1.021	0.164	2.206	d_9_	3.295	2.239	1.056	5.534
					d_10_	2.640	1.870	0.770	4.510
					d_11_	2.005	1.537	0.468	3.542
D_4_	1.182	1.348	−0.166	2.530	d_12_	3.171	3.551	−0.380	6.722
					d_13_	2.924	3.561	−0.637	6.485
					d_14_	3.145	3.877	−0.732	7.022
					d_15_	2.853	3.846	−0.993	6.699
D_5_	1.171	1.301	−0.130	2.472	d_16_	3.209	3.764	−0.555	6.973
					d_17_	3.058	3.788	−0.730	6.846
					d_18_	2.720	3.531	−0.811	6.251
					d_19_	2.767	3.635	−0.868	6.402
					d_20_	3.166	3.409	−0.243	6.575

Here, (*f*_*i*_−*e*_*i*_)is the difference between the influences given by factor *i* to other factors and those received by it from other factors, reflecting the degree of influence of factor *i* on other factors in the system. For instance, (*f*_*i*_+*e*_*i*_) is the sum of influences given and received by factor *i*; *f*_*i*_−*e*_*i*_>0 indicates that factor *i* has an influence on other factors in the system; whereas *f*_*i*_−*e*_*i*_ <0 indicates that factor *i* is influenced by other factors in the system.

### Calculating the Influence Weights of Dimensions and Factors

The weights of the influences between dimensions and factors were calculated using DANP. The steps are as follows:

Step 1: Build an unweighted matrix. The total relation matrices based on dimensions and factors are represented by $T_{d}={\left[t_{ij}^{D}\right]}_{m\times m}$ and *T*_*c*_ =_[*t*_*ij*_]*n*×*n*_, respectively. *T*_*c*_ is expressed by equation (9):


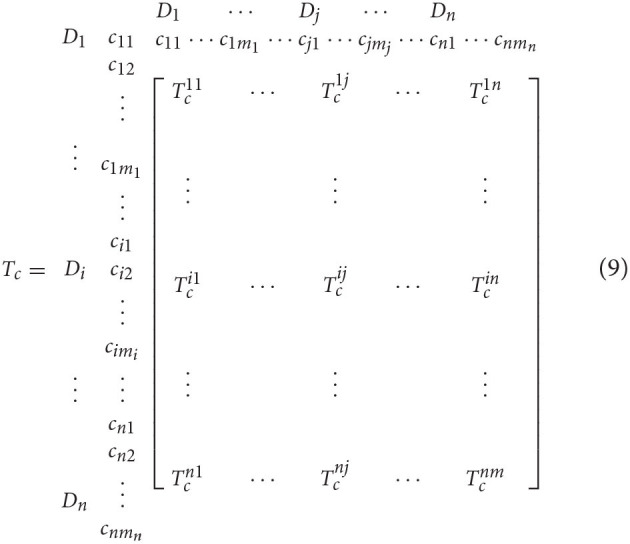


The normalized total relation matrix $T_{c}^{\alpha }$ was obtained by normalizing *T*_*c*_. The normalization process is illustrated by taking the submatrix $T_{c}^{11}$ of equation (10) as an example. The row sum of elements in row *i* in $T_{c}^{11}$ is denoted as $d_{i}^{11}$.


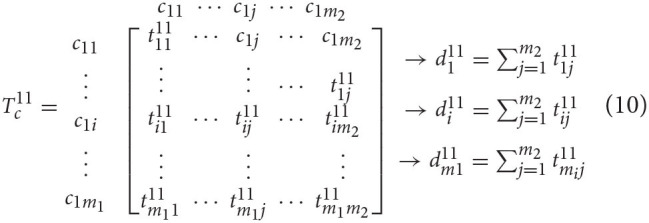


Each element in row *i* of the matrix was divided by the sum of row *i*, and then the following normalized submatrix $T_{c}^{\alpha 11}$ was obtained:


(11) $$\begin{array}{r} T_{c}^{\alpha 11}=\left[\begin{array}{ccccc} \frac{t_{11}^{11}}{d_{1}^{11}} & \cdots & \frac{t_{1j}^{11}}{d_{1}^{11}} & \cdots & \frac{t_{1m_{2}}^{11}}{d_{1}^{11}}\\ \vdots & \vdots & \vdots \\ \frac{t_{i1}^{11}}{d_{i}^{12}} & \cdots & \frac{t_{ij}^{11}}{d_{i}^{12}} & \cdots & \frac{t_{im_{2}}^{11}}{d_{i}^{12}}\\ \vdots & \vdots & \vdots \\ \frac{t_{m_{1}1}^{11}}{d_{m_{1}}^{11}} & \cdots & \frac{t_{m_{1}j}^{11}}{d_{m_{1}}^{11}} & \cdots & \frac{t_{m_{1}m_{2}}^{11}}{d_{m_{1}}^{11}} \end{array}\right]=\left[\begin{array}{ccccc} t_{11}^{\alpha 11} & \cdots & t_{1j}^{\alpha 11} & \cdots & t_{1m_{2}}^{\alpha 11}\\ \vdots & \vdots & \vdots \\ t_{i1}^{\alpha 11} & \cdots & t_{ij}^{\alpha 11} & \cdots & t_{im_{2}}^{\alpha 11}\\ \vdots & \vdots & \vdots \\ t_{m_{1}1}^{\alpha 11} & \cdots & t_{m_{1}j}^{\alpha 11} & \cdots & t_{m_{1}m_{2}}^{\alpha 11} \end{array}\right] \end{array}$$

The unweighted supermatrix *W* was obtained by transposing each normalized submatrix, where $W_{ij}={\left(T_{c}^{\alpha ji}\right)}^{T},i=1,2\ldots ,n,j=1,2,\ldots \;n$.


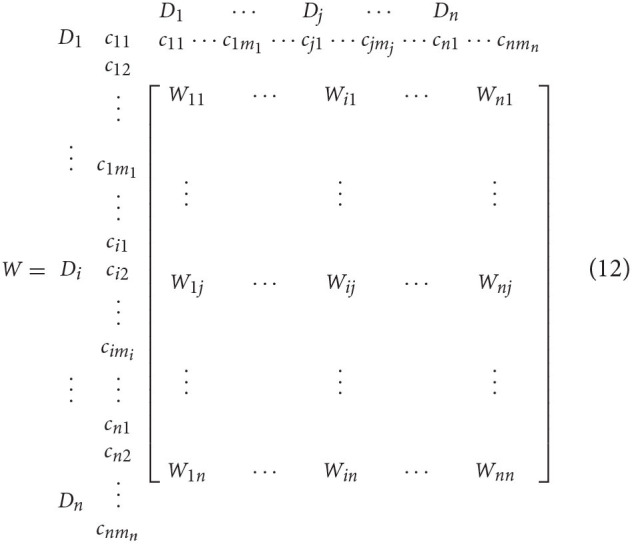


Step 2: Build the weighted supermatrix $t_{ij}^{D}$. Similar to the method used for processing the total relation matrix *T*_*c*_ based on the influencing factors, the total relation matrix *T*_*d*_ between dimensions was normalized to obtain the matrix $T_{d}^{\alpha }$:


(13) $$\begin{array}{r} T_{d}^{\alpha }=\left[\begin{array}{ccccc} \frac{t_{11}^{d}}{d_{1}} & \cdots & \frac{t_{1j}^{d}}{d_{1}} & \cdots & \frac{t_{1n}^{d}}{d_{1}}\\ \vdots & \vdots & \vdots \\ \frac{t_{i1}^{d}}{d_{i}} & \cdots & \frac{t_{ij}^{d}}{d_{i}} & \cdots & \frac{t_{in}^{d}}{d_{i}}\\ \vdots & \vdots & \vdots \\ \frac{t_{n1}^{d}}{d_{n}} & \cdots & \frac{t_{nj}^{d}}{d_{n}} & \cdots & \frac{t_{nn}^{d}}{d_{n}} \end{array}\right]=\left[\begin{array}{ccccc} t_{11}^{\alpha d} & \cdots & t_{1j}^{\alpha d} & \cdots & t_{1n}^{\alpha d}\\ \vdots & \vdots & \vdots \\ t_{i1}^{\alpha d} & \cdots & t_{ij}^{\alpha d} & \cdots & t_{in}^{\alpha d}\\ \vdots & \vdots & \vdots \\ t_{n1}^{\alpha d} & \cdots & t_{nj}^{\alpha d} & \cdots & t_{nn}^{\alpha d} \end{array}\right] \end{array}$$

The unweighted matrix *W* and dimension weight matrix of factors affecting public-public collaboration were calculated using equation (14) to obtain the weighted supermatrix *W*_*w*_:


(14) $$\begin{array}{r} W_{w}=T_{d}^{\alpha }\times W=\left[\begin{array}{ccccc} t_{11}^{\alpha d}\times W_{11} & \cdots & t_{i1}^{\alpha d}\times W_{1i} & \cdots & t_{n1}^{\alpha d}\times W_{1n}\\ \vdots & \vdots & \vdots \\ t_{1i}^{\alpha d}\times W_{i1} & \cdots & t_{ii}^{\alpha d}\times W_{ii} & \cdots & t_{ni}^{\alpha d}\times W_{in}\\ \vdots & \vdots & \vdots \\ t_{1n}^{\alpha d}\times W_{n1} & \cdots & t_{in}^{\alpha d}\times W_{ni} & \cdots & t_{nn}^{\alpha d}\times W_{nn} \end{array}\right] \end{array}$$

Step 3: Calculate the limit supermatrix for weights. The weighted supermatrix *W*_*w*_ was exponentiated until it converged to a stable state. When the elements in each row of the weighted supermatrix are the same, the matrix reaches a stable state. At this point, the limit supermatrix W^*^ shown in equation (15) was obtained, and the weight of each influencing factor was determined.


(15) $$\begin{array}{r} W^{*}=\underset{k\;\to \;\infty }{\text{lim}}W_{w}^{k} \end{array}$$

Based on the literature review and the actual situation of public-public collaboration for food safety risk management in China, a set of factors that affect public-public collaboration in the global context was constructed by the five dimensions, i.e., basic characteristics, legal basis, functions, behavior and capabilities, and infrastructure and culture of government agencies. First, the total relation matrix was divided by the set of influencing factors. Then, the resulting submatrices were normalized and transposed to obtain the unweighted supermatrix of factors affecting public-public collaboration for food safety risk management. The unweighted matrix and dimension weights of the influencing factors were calculated to obtain the weighted supermatrix *W*^α^ of the influencing factors. The limit of the weighted supermatrix was programed and calculated using MATLAB. The weighted supermatrix *W*^α^ was exponentiated until the results converged to the stable limit supermatrix W^*^ shown in Table 5.

**Table 5:** Limit supermatrix of influencing factors.

**Factors**	**d_**1**_**	**d_**2**_**	**d_**3**_**	**d_**4**_**	**d_**5**_**	**d_**6**_**	**d_**7**_**	**d_**8**_**	**d_**9**_**	**d_**10**_**
d_1_	0.038	0.038	0.038	0.038	0.038	0.038	0.038	0.038	0.038	0.038
d_2_	0.021	0.021	0.021	0.021	0.021	0.021	0.021	0.021	0.021	0.021
d_3_	0.020	0.020	0.020	0.020	0.020	0.020	0.020	0.020	0.020	0.020
d_4_	0.053	0.053	0.053	0.053	0.053	0.053	0.053	0.053	0.053	0.053
d_5_	0.047	0.047	0.047	0.047	0.047	0.047	0.047	0.047	0.047	0.047
d_6_	0.090	0.090	0.090	0.090	0.090	0.090	0.090	0.090	0.090	0.090
d_7_	0.115	0.115	0.115	0.115	0.115	0.115	0.115	0.115	0.115	0.115
d_8_	0.099	0.099	0.099	0.099	0.099	0.099	0.099	0.099	0.099	0.099
d_9_	0.048	0.048	0.048	0.048	0.048	0.048	0.048	0.048	0.048	0.048
d_10_	0.040	0.040	0.040	0.040	0.040	0.040	0.040	0.040	0.040	0.040
d_11_	0.033	0.033	0.033	0.033	0.033	0.033	0.033	0.033	0.033	0.033
d_12_	0.048	0.048	0.048	0.048	0.048	0.048	0.048	0.048	0.048	0.048
d_13_	0.048	0.048	0.048	0.048	0.048	0.048	0.048	0.048	0.048	0.048
d_14_	0.050	0.050	0.050	0.050	0.050	0.050	0.050	0.050	0.050	0.050
d_15_	0.052	0.052	0.052	0.052	0.052	0.052	0.052	0.052	0.052	0.052
d_16_	0.041	0.041	0.041	0.041	0.041	0.041	0.041	0.041	0.041	0.041
d_17_	0.042	0.042	0.042	0.042	0.042	0.042	0.042	0.042	0.042	0.042
d_18_	0.038	0.038	0.038	0.038	0.038	0.038	0.038	0.038	0.038	0.038
d_19_	0.039	0.039	0.039	0.039	0.039	0.039	0.039	0.039	0.039	0.039
d_20_	0.037	0.037	0.037	0.037	0.037	0.037	0.037	0.037	0.037	0.037
**Factors**	**d**_**11**_	**d**_**12**_	**d**_**13**_	**d**_**14**_	**d**_**15**_	**d**_**16**_	**d**_**17**_	**d**_**18**_	**d**_**19**_	**d**_**20**_
d_1_	0.038	0.038	0.038	0.038	0.038	0.038	0.038	0.038	0.038	0.038
d_2_	0.021	0.021	0.021	0.021	0.021	0.021	0.021	0.021	0.021	0.021
d_3_	0.020	0.020	0.020	0.020	0.020	0.020	0.020	0.020	0.020	0.020
d_4_	0.053	0.053	0.053	0.053	0.053	0.053	0.053	0.053	0.053	0.053
d_5_	0.047	0.047	0.047	0.047	0.047	0.047	0.047	0.047	0.047	0.047
d_6_	0.090	0.090	0.090	0.090	0.090	0.090	0.090	0.090	0.090	0.090
d_7_	0.115	0.115	0.115	0.115	0.115	0.115	0.115	0.115	0.115	0.115
d_8_	0.099	0.099	0.099	0.099	0.099	0.099	0.099	0.099	0.099	0.099
d_9_	0.048	0.048	0.048	0.048	0.048	0.048	0.048	0.048	0.048	0.048
d_10_	0.040	0.040	0.040	0.040	0.040	0.040	0.040	0.040	0.040	0.040
d_11_	0.033	0.033	0.033	0.033	0.033	0.033	0.033	0.033	0.033	0.033
d_12_	0.048	0.048	0.048	0.048	0.048	0.048	0.048	0.048	0.048	0.048
d_13_	0.048	0.048	0.048	0.048	0.048	0.048	0.048	0.048	0.048	0.048
d_14_	0.050	0.050	0.050	0.050	0.050	0.050	0.050	0.050	0.050	0.050
d_15_	0.052	0.052	0.052	0.052	0.052	0.052	0.052	0.052	0.052	0.052
d_16_	0.041	0.041	0.041	0.041	0.041	0.041	0.041	0.041	0.041	0.041
d_17_	0.042	0.042	0.042	0.042	0.042	0.042	0.042	0.042	0.042	0.042
d_18_	0.038	0.038	0.038	0.038	0.038	0.038	0.038	0.038	0.038	0.038
d_19_	0.039	0.039	0.039	0.039	0.039	0.039	0.039	0.039	0.039	0.039
d_20_	0.037	0.037	0.037	0.037	0.037	0.037	0.037	0.037	0.037	0.037

Based on the calculation results of the limit matrix, the relative weights of each factor interacting in the whole factor set as shown in Table 6 were obtained.

**Table 6:** Weights and ranking of factors affecting public-public collaboration for food safety risk management.

**Object**	**Dimensions**	**Weights of dimensions**	**Factors**	**Weights of factors**	**Ranking**
Public-Public			d_1_	0.038	15
collaboration for Food	D_1_	0.133	d_2_	0.021	19
Safety Risk			d_3_	0.020	20
Management			d_4_	0.053	4
			d_5_	0.047	10
	D_2_	0.351	d_6_	0.090	3
			d_7_	0.115	1
			d_8_	0.099	2
			d_9_	0.048	7
	D_3_	0.121	d_10_	0.040	13
			d_11_	0.033	18
			d_12_	0.048	7
	D_4_	0.197	d_13_	0.048	7
			d_14_	0.050	6
			d_15_	0.052	5
			d_16_	0.041	12
			d_17_	0.042	11
	D_5_	0.198	d_18_	0.038	15
			d_19_	0.039	14
			d_20_	0.037	17

### Analysis of Calculation Results

According to the above calculation results, the following analysis was conducted:

#### Interrelationships Between Dimensions and Identification of Key Dimensions

**Figure 1:**
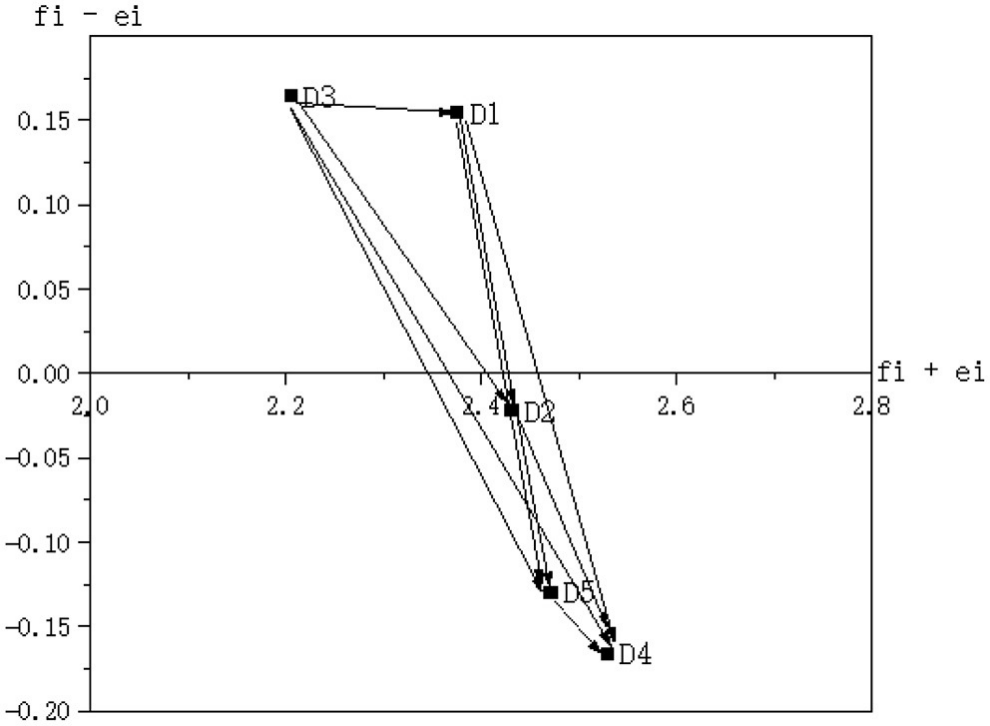
Influence relation map of dimensions affecting public-public collaboration for food safety risk management.

**Figure 2:**
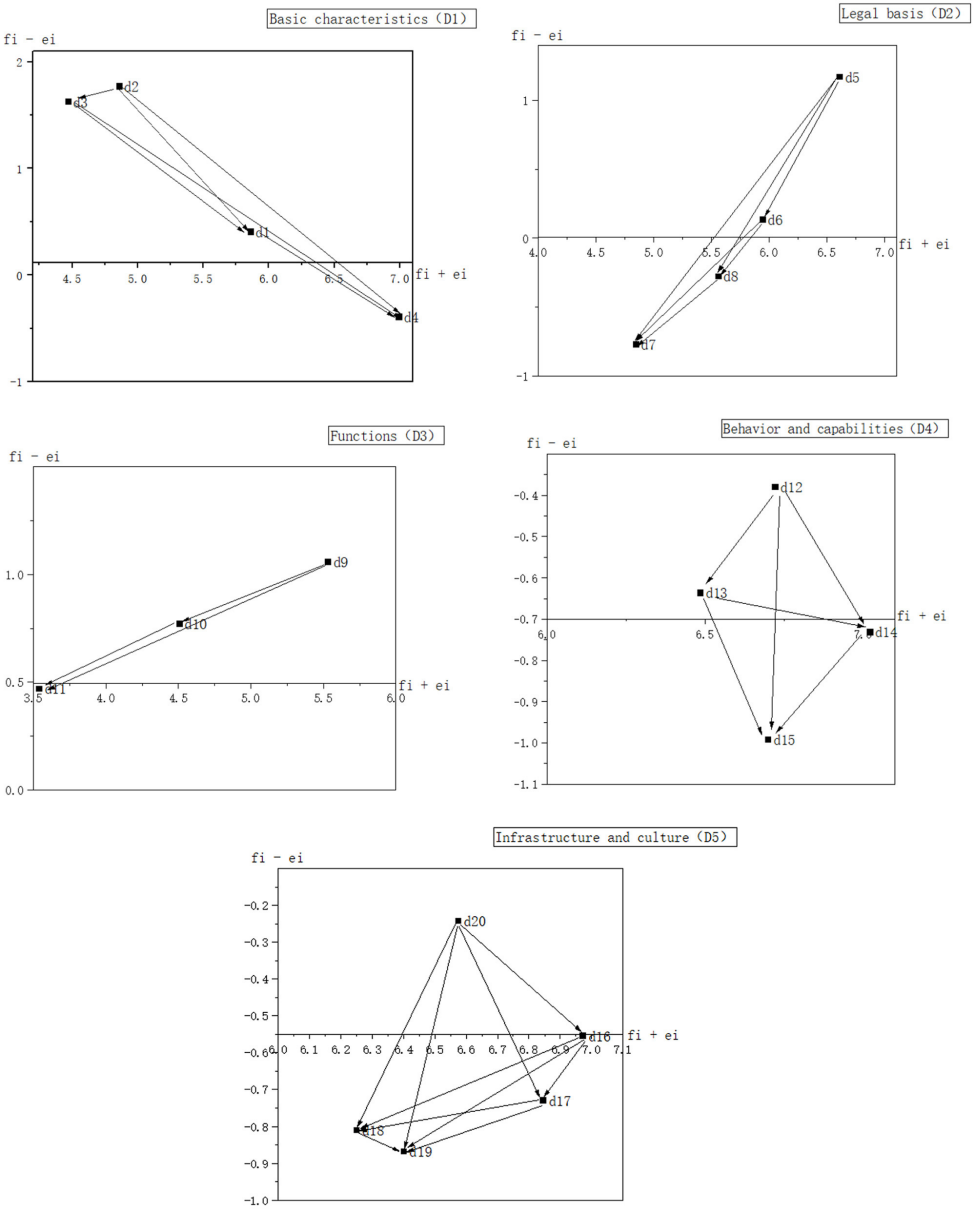
Influence relation map of factors affecting public-public collaboration for food safety risk management.

According to the data set (*f*_*i*_−*e*_*i*_, *f*_*i*_+*e*_*i*_) for DEMATEL in Table 4, network diagrams of the relationships between the five dimensions and 20 factors were created as shown in Figures 1, 2, respectively.

According to Chiu et al. (73), the value of (*f*_*i*_−*e*_*i*_) is the main basis for determining the relationship between dimensions and identifying the cause and effect dimensions. Therefore, the relationships between dimensions and factors that affect public-public collaboration for food safety risk management can be analyzed based on the values of (*f*_*i*_−*e*_*i*_) calculated in Table 4. As shown in Table 4, the (*f*_*i*_−*e*_*i*_)values of D_1_ and D_3_, i.e., 0.154 and 0.164, are positive, indicating that D_1_ and D_3_ more actively affect other dimensions in the system and thus can be identified as the cause dimensions. The values of (*f*_*i*_−*e*_*i*_) of D_2_, D_4_, and D_5_, i.e., −0.022, −0.166, and −0.130, are negative, indicating that D_2_, D_4_, and D_5_ are more affected by other dimensions in the system and thus can be identified as the result dimension.

Figure 1 shows that the influence relationship between the five dimensions is D_3_ → D_1_ → D_2_ → D_5_ → D_4_, D_1_ → D_2_ → D_5_ → D_4_, D_2_ → D_5_ → D_4_, and D_5_ → D_4_ (where → indicates the direction of influence; the same definition is used below). These findings suggest that among the five dimensions affecting public-public collaboration for food safety risk management, D_3_ has the greatest influence on the other four dimensions, and D_4_ is most affected by the other four dimensions. D_1_, D_2_, and D_5_ are both affected by and affect other dimensions, but the influences they received are greater than those they have over other dimensions. This result also suggests that the first step to achieve public-public collaboration for food safety risk management is to clearly define the functions of government agencies. It is necessary to minimize the blurring of functional boundaries and clarify their basic role types (comprehensive dominant, single-function dominant, or auxiliary) and the power level, subordination, and professionalism.

Secondly, the basis for implementing public-public collaboration should be recognized by laws, regulations, and normative documents, etc.; the agency behavior and capabilities should be specified and regulated; and the infrastructure and culture should be improved to better support public-public collaboration.

According to Huang et al. (69), the size of influence weight and the positive or negative value of causality are the main basis for identifying key dimensions. Further analysis can be performed based on the data in Table 6. Among the system's five dimensions, D_2_ has the highest influence weight (0.351), indicating that it has the greatest influence in the system of public-public collaboration for food safety risk management. Although its value of (*f*_*i*_−*e*_*i*_) is −0.022, which is negative, it tends to 0, indicating that the influences it receives from the other four dimensions also tend to 0. Indeed, this is not difficult to understand because the basic characteristics, functions, behavior and capabilities, and infrastructure and culture of government agencies are all derived from the legal basis. The legal basis is the most authoritative and binding, and is stable in the long term. Therefore, it can be determined that the legal basis is a key dimension that plays a fundamental role among all dimensions.

#### Interrelationships Between Factors Affecting Public-Public Collaboration

The values of (*f*_*i*_−*e*_*i*_) in Table 4 reflect the degree of mutual influence among the system's 20 factors. Although these factors influence each other directly or indirectly, d_2_ has the largest value of (*f*_*i*_−*e*_*i*_) of 1.77 among all factors. It suggests that this factor has the greatest causality but varying degrees of influence on all other factors in the system, and thus can be identified as a cause factor. In contrast, d_15_ has the smallest value of (*f*_*i*_−*e*_*i*_) (−0.243) among all factors, indicating that this factor has the smallest causality and is more affected by other factors in the system, and thus can be identified as an effect factor.

Figure 2 depicts the direct influence relationships between the factors that constitute the five dimensions. The values of (*f*_*i*_−*e*_*i*_) of the four factors d_1_, d_2_, d_3_, and d_4_ that constitute D_1_ are 0.404, 1.770, 1.625, and −0.395, respectively. Among them, d_2_ has the largest value and thus can be considered the most influential factor in D_1_. Hence, it can be concluded that the influence relationship between the four characteristic factors is as follows: d_2_ → d_3_ → d_1_ → d_4_, d_3_ → d_1_ → d_4_, and d_1_ → d_4_. Likewise, the influence relationships between factors in the other four dimensions can be expressed in the same way as for those in D_1_ (Figure 2). As shown in Table 4, with the values of (*f*_*i*_−*e*_*i*_) being 1.170, 1.056, −0.380, and −0.243, d_5_, d_9_, d_12_, and d_20_ are identified as the most influential factors in D_2_, D_3_, D_4_, and D_5_, respectively.

#### Identification of Key Factors Affecting Public-Public Collaboration

Table 6 presents the influence weights of the five dimensions and 20 factors affecting public-public collaboration for food safety risk management. Different criteria are needed for identifying key factors in a complex system composed of multiple factors. In general, taking into consideration the specific characteristics of the research object, key factors are identified in DANP, with the values of (*f*_*i*_+*e*_*i*_) as the main criterion and the influence weight as the auxiliary criterion (74). Among the 20 factors in this system, d_14_ has the highest value of (*f*_*i*_+*e*_*i*_) (7.022) and ranks sixth in terms of influence weight, indicating that this factor has the greatest combined influence on other factors. It demonstrates that the principle that “government must carry out all statutory functions and duties and may not do anything not authorized by law” plays an extremely important fundamental role in public-public collaboration. Hence, d_14_ is identified as a key factor. This is highly consistent with the conclusion of Gazley (57) that government agencies should implement public-public collaboration through legislative authorization.

The (*f*_*i*_+*e*_*i*_) value of d_4_ (6.993) ranks second and its influence weight ranks fourth, meaning that it can also be identified as a key factor. This is not only strikingly consistent with the actual situation of food safety risk management in China and Western countries, but also agrees with the findings of Woldesenbet (35). The (*f*_*i*_+*e*_*i*_) value of d_12_ (6.722) ranks fifth, and its influence weight ranks seventh, so that it can also be identified as a key factor. This is because the effect of public-public collaboration among government agencies is determined by their comprehensive administrative capabilities, such as decision-making, execution, and coordination. This finding is consistent with the conclusion of Boatemaa et al. (55).

The (*f*_*i*_+*e*_*i*_) value of d_15_ (6.699) ranks sixth, and its influence weight ranks fifth, indicating that food safety risk management depends on the improvement of the entire social environment and thus requires indirect participation by government agencies that perform auxiliary functions. This is consistent with the findings of Karp et al. (59). Hence, it can be identified as a key factor. The (*f*_*i*_+*e*_*i*_) value of d_5_ (6.614) ranks seventh, and its influence weight ranks tenth, indicating that this factor also exerts a certain influence on the system. This is because the legalization and institutionalization of government agencies' functions serves as a fundamental guarantee for the performance of their governance functions. This result is consistent with the conclusion of Koebele (41). Hence, it can also be identified as a key factor. To sum up, these are the five key factors affecting the governance capacity in public-public collaboration for food safety risk management.

It should be pointed out that although the (*f*_*i*_+*e*_*i*_) values of d_16_ and d_17_ (6.973 and 6.864) rank third and fourth, respectively, their influence weights are relatively low, ranking twelfth and thirteenth, respectively. Therefore, it can be concluded that they have relatively limited combined influences and therefore should not be identified as key factors. Nevertheless, the ranking results do suggest that promoting information sharing and information technology is also important for public-public collaboration.

## Conclusions, Implications for China, and Prospects

### Conclusions

Three conclusions can be drawn from the findings obtained from this study. First, public-public collaboration is affected by many dimensions and factors that interweave and interact with each other and form a complex system. Second, among the five dimensions, the legal basis has the highest influence weight, is the most critical dimension, and inherently affects the other four dimensions. This is because the principle that “government must carry out all statutory functions and duties and may not do anything not authorized by law” is a fundamental criterion for government agencies in modern society to have when performing their functions.

Third and lastly, among the 20 influencing factors, legislation-based governance, professionalism of government agencies, administrative law enforcement–based governance, social environment improvement–based governance, and having laws and regulations as legal basis are the most important factors. This is because government agencies implement public-public collaboration through legislative authorization. Moreover, because food safety risk management has high professional requirements, it is necessary to implement administrative law enforcement governance under the framework of legal authorization. Although informal rules are also valuable, public-public collaboration relies more on laws and regulations.

### Implications for China

The conclusions of this study provide important guidance to the Chinese government. First, based on the essential requirements of food safety risk management in modern society, the capacity-building of legislative bodies should be strengthened to solve the problems of fragmentation of legislation and contradictions between laws to form a complete and interconnected system of laws and regulations. Efforts should also be made to improve the law enforcement environment and overcome local protectionism.

Second, although it is impossible to change the practice of using normative documents formulated by the government as a legal basis in the Chinese context, normative documents should be designed to minimize gaps and ambiguity in the legal system. It is also necessary to solve the problems of excessive and contradictory normative documents, and to abolish outdated normative documents in a timely manner to ensure that laws are not affected by normative documents.

Third, a hierarchical governance system for food safety management composed of government agencies with clear powers and responsibilities on the one hand and complementary functions from the central to the local levels on the other hand should be developed to solve the persistent problems in public-public collaboration, such as blurred boundaries, gaps, and fragmentation.

Fourth, efforts should be made to strengthen the professional development of government agencies to build a professional talent team and ensure adequate technical facilities. Fifth and lastly, public-public collaboration for food safety risk management depends on the improvement of the entire social environment. It not only requires the efforts of dominant government agencies, including market regulation, agriculture and rural affairs, customs, and health agencies, but also the participation of auxiliary agencies such as those responsible for commerce, grain, forestry, and education. The above suggestions, especially building a complete legal system, strengthening the capacity-building of professional institutions, accelerating the adoption of information technology and infrastructure construction for food safety information systems, and promoting information flows among government agencies, also have implications for many developing countries.

### Prospects

However, there are limitations in this study. The original data used in DANP were drawn from a Chinese expert group instead of a representative group of experts from different countries. The importance assigned to each dimension and factor largely depended on the observation or understanding of the situation of China by these Chinese experts. Moreover, Chinese characteristics were considered when determining the key dimensions and factors affecting public-public collaboration. For example, the use of government-issued normative documents (d_6_) as a legal basis is apparently characteristic of China.

Therefore, the global applicability of this study's conclusions needs to be further assessed. Nevertheless, this study provides an approach for the academic community to use to understand the main problems facing public-public collaboration for food safety risk management in China, and it provides decision-making support for the Chinese government to promote public-public collaboration. Moreover, this study also has implications for many developing countries, especially that the government cannot simply replace laws with executive orders.

## Data Availability Statement

The raw data supporting the conclusions of this article will be made available by the authors, without undue reservation.

## Author Contributions

All authors undertook research, writing, review tasks throughout this study, read, and agreed to the published version of the manuscript.

## Funding

This work was supported by the National Social Science Fund of China: Research on Social Co-governance of Food Safety Risks and Cross-Border Cooperative Governance Mechanism (Grant No. 20&ZD117).

## Conflict of Interest

The authors declare that the research was conducted in the absence of any commercial or financial relationships that could be construed as a potential conflict of interest.

## Publisher's Note

All claims expressed in this article are solely those of the authors and do not necessarily represent those of their affiliated organizations, or those of the publisher, the editors and the reviewers. Any product that may be evaluated in this article, or claim that may be made by its manufacturer, is not guaranteed or endorsed by the publisher.
